# Supplementary material for: Detection of traces of calcium oxalate druses in fossil leaves of angiosperms and gymnosperms from different sites and geological periods
Source: PLoS One. 2025 Dec 17;20(12):e0332423. doi: 10.1371/journal.pone.0332423 (PMC12711041; doi:10.1371/journal.pone.0332423)
Supplement: Attachment — Submitted filename: letter with response to reviewers comments.docx [file pone.0332423.s001.docx]

**Letter to the Reviewers' comments to the below manuscript:**

PONE-D-23-19584
“The evolutionary history and fossil record of calcium oxalate druses in fossil leaves of angiosperms and gymnosperms”

which are explained in detail in red colour and in the track change version in yellow and blue to answer the reviewers 1 and 2 respectively.

Reviewer's Responses to Questions

**Comments to the Author**

1. Is the manuscript technically sound, and do the data support the conclusions?

The manuscript must describe a technically sound piece of scientific research with data that supports the conclusions. Experiments must have been conducted rigorously, with appropriate controls, replication, and sample sizes. The conclusions must be drawn appropriately based on the data presented.

Reviewer #1: Yes

Reviewer #2: Partly

2. Has the statistical analysis been performed appropriately and rigorously?

Reviewer #1: N/A

Reviewer #2: N/A

3. Have the authors made all data underlying the findings in their manuscript fully available?

The [PLOS Data policy](http://www.plosone.org/static/policies.action#sharing) requires authors to make all data underlying the findings described in their manuscript fully available without restriction, with rare exception (please refer to the Data Availability Statement in the manuscript PDF file). The data should be provided as part of the manuscript or its supporting information, or deposited to a public repository. For example, in addition to summary statistics, the data points behind means, medians and variance measures should be available. If there are restrictions on publicly sharing data—e.g. participant privacy or use of data from a third party—those must be specified.

Reviewer #1: Yes

Reviewer #2: Yes

4. Is the manuscript presented in an intelligible fashion and written in standard English?

PLOS ONE does not copyedit accepted manuscripts, so the language in submitted articles must be clear, correct, and unambiguous. Any typographical or grammatical errors should be corrected at revision, so please note any specific errors here.

Reviewer #1: Yes

Reviewer #2: Yes

5. Review Comments to the Author

Please use the space provided to explain your answers to the questions above. You may also include additional comments for the author, including concerns about dual publication, research ethics, or publication ethics. (Please upload your review as an attachment if it exceeds 20,000 characters)

**Reviewer #1:** The study examined angiosperm and gymnosperm fossils from different ages and sites and found granular structures of various morphology and composition. These granular structures were interpreted as CaOx druses or crystals. The possible function and preservational pattern of these possible CaOx druses or crystals have been discussed.
This work provides plenty of new data about the microstructures of fossil leaves. But the data are scattered among different species, ages, and sedimentary backgrounds. In my view, a definite conclusion, a clearer summary of the preservational pattern, and more words to support the hypotheses in the discussion part would improve the paper.

Major points:
Abstract
1. Page 2, Line 22-23. The previously reported CaOx traces in Oligocene dicotyledon are referred to, but the problems or questions this paper deals with are not stated. Something like "But there is still no certain report of CaOx druses traces in Paleozoic or Mesozoic fossils" can be added.

Reply: Yes; done.

2. Page 2, Line 9-15 or Page 3 Line 1-6. These three sentences show the paper's results in terms of species, ages, and composition. The three aspects of the pattern can be stressed, and the exact pattern needs to be stated. Modification can be like: "We found granular structures that can be interpreted as casts of CaOx druses are not limited to certain clades, ages, or sites. Angiosperm fossils from various sites, seed ferns since Devonian, and Ginkgophytae since Carboniferous all can preserve traces of CaOx druses. But the composition of these CaOx casts varies according to the chemistry of the fossils and the surrounding matrix (the relationship needs to be further summarized)".

Yes; we used this alternative text.

3. Page 3, Line 4-6. "Identification of the biomineral traces is important to help classify or reclassify fossil plants, to reconstruct the evolutionary history of plants…". The cases in this paper do not clarify these two aspects of importance. You can add your conclusions about the preservational pattern here.

Introduction

4. Page 3-6. The paragraph sequence can be better if the text introduces fresh dicots (the original first paragraph on Page 3), monocots (the last sentence on Page 4), and gymnosperms (the initial first paragraph on Page 6) to show the diverse forms and characteristic shapes and sizes, then talks about the scarce fossil records (the first paragraph in Page 4, the second paragraph in Page 4 except the last sentence).

Yes; we re-arranged the paragraphs.

5. Page 6. Before "In the present study we examined…" a statement of the current problem (e.g., "the lack of fossil records limits our understanding of the evolution of CaOx and associated ecological behaviour in plants") may help to stress the motivation of the study.

Ok; included.

Results

6. Page 12, Line 22. "Gymnosperms have simpler or less variable patterns". You can specify the patterns (including the exact morphology, size, and distribution) here, in addition to the qualified "large" or "small" in the following sentence.

The patterns are specified in the following sentences. ‚Sizes‘ are added.

7. Page 12-13. The first paragraph of the Results seems to be about the identification methods. The first two sentences in the third paragraph of Results ("The fossil samples from…the Institutions or Museums") seem to be about the study materials. Maybe you can consider moving them into the Materials and Methods section.

Yes; we have re-arranged this part and included it into Materials and Methods.

8. The morphological and distributional similarities between the granular structures and CaOx druses of fresh leaves can be stated more clearly and stressed in each case. These are the evidence of the interpretation and essential bases for the following discussion about the pattern. For example, on Page 14, Line 13, the relationship between "this distribution" (which distribution?) and "been substituted" is not clear. You can consider modification of these two sentences, like "the regular distribution is similar to the CaOx druses in fresh leaves, e.g., in xxx, which support the interpretation of these pyrite-rich granules as CaOx traces. EDS analyses reveal the composition of granules…The pyrite-rich chemistry suggests that these druses have been substituted during fossilization with available elements of the surrounding environment".

Page 14 Line 13: ok; paragraph written more precisely.

9. Figures 3 and 6. You can consider adding overview photos of the fossil specimens to support the classification.
10. Page 24, Line 22. No figures or references show the "dark brown granules".

We have marked the granules in Fig. 8F.

Discussion
11. Page 26. Line 18. This paragraph about fossil records should be connected to the defense hypothesis in the previous paragraph.

Ok; paragraph shifted to the right place.

12. Page 26, Line 22. This paragraph can be separated into two, one about calcium metabolism and one about hardness and stability. Can the fossil results in this paper support some of these functional hypotheses?

We hope, and it seems, that the fossil results are in accordance with results from extant plants. However, we think that the main benefit of our result is the identification and correct interpretation of the granular structures, which were formerly not recognized.

13. Page 27, Line 16. "Which are apparently arranged along the leaf venation". This pattern is unclear in Fig. 6D. You can consider marking the venation.

Ok; venation patterns is marked with arrows in Fig. 6D.

14. The "Fossil record of CaOx cast preservation" section does not provide much more information than the Results section. You can consider making a table to summarize these granular structures' distribution, morphology, and chemistry from different species, ages, and localities.

Ok; we have deleted most of the redundant information and re-arranged the Discussion.

15. Page 29, Lines 6-7. "Differences in fossilization conditions in various sites". This aspect of information was not shown in the paper. You can consider adding this information to a summarizing table.

‚the most influencial and limiting‘ deleted; so the statement seems to be correct.

16. Page 29, Line 22. It would help to have evidence and references for the "leaves with thicker cuticle … is significantly better than in other leaves" conclusion.

‚preservation‘ replaced by ‚identification is more reliable‘.

17. Page 30, Line 1. It would help if you had evidence and references supporting the effect of "cuticle thickness" for preservation.

This sentence deleted.

18. Page 30, Line 10. "More experience and more data are necessary to identify". This sentence is not informative or relevant to the preservational pattern.

This statement seems relevant in the context of emerging techniques like pattern recognition.

Minor points:
Abstract
1. The abstract is mainly duplicated. The second version is better.

Duplicate deleted.

2. Page 2, Line 22, "here the CaOx was replaced". "here" may cause misunderstanding that this clause is talking about the current paper. "In these leaves, the CaOx was replaced" may be better.

Yes; replaced.

3. Page 2, Line 25-26, "which were compared to CaOx druses in extant gymnosperm species". This clause is suggested to be moved behind "granular structures in carbonized or mineralized leaf remnants" in Line 11. The species and ages are the core topic of this sentence.

Ok, done.

4. Page 3, Line 3. "Scanning electron microscopy revealed varying compositions". Listing the several types of "varying composition" (pyrites, organic, silica, etc.) may be better.

Ok; inserted.

Introduction
5. Page 4, Line 9. "But they were highly variable in composition" needs a reference.

Ok; included.

6. Page 6, Line 12. "In leaves of Ginkgo biloba this process is documented in form of very large druses (Raman& Croom, 1998)." "This process" needs to be clarified.

Ok; replaced by ‚the presence of CaOx‘

7. Page 6, Line 14. "Their characteristic distribution patterns." You can consider clarifying the pattern here (e.g., along venation or regularly distributed in mesophyll).

Ok; done.

Materials and Methods
8. Page 11, Line 1. The parameters of the SEM observation, such as voltage and working distances, are helpful if available.

Included.

9. Page 11, Line 15. You can consider specifying the instrument for sputter-coating.
10. Page 11, Line 20-21. This paragraph is about fossil samples. The sputter-coating methods for "fresh leaves" should be moved to the next paragraph.

Ok; done.

Results
11. Page 13, Line 11. "The leaf lamina contains druses in varying density". You can clarify how the druse density is "varying".

‚Density‘ replaced by ‚concentration‘.

12. Page 13, Line 22. "Because druses usually occur in the leaf parenchym(a), but rarely in the epidermis" needs a reference.

Ok; included.

13. Page 14, Line 7. "Iron oxide caused by storage in glycerol" needs a reference.

Additional information or images on these contamination seem unnecessary and confusing.

14. Page 14, Line 13. The scientific name "Hedera helix" should be in italics.

Ok; done

15. Page 16, Line 19-21. “This formation…Røedvika Formation (Horn & Orvin, 1928)”. The stratigraphy study's history is irrelevant to the specimen's age and the paper's topic.
16. Page 16, Line 24. "Thus" seems strange as the locality of the fossils should be known rather than gotten from deduction.

Ok; deleted.

17. Page 17, Line 1. It is unclear what subject "which" represents, the fragments or the pyrite particles.

It is clear by following term ‚leaf surface structures‘.

18. Page 17, Line 5. You can consider specifying what "shape" and what "location" the granular pyrite inclusions have.

done

19. Page 19, Line 5. "Resembled those of CaOx druses in fresh leaves" need references.

Done; references included.

20. Page 20, Line 11. "A distribution pattern that resembles the distribution of CaOx druses". You can specify what kind of pattern they have.

Done; included ‚in parallel rows‘.

21. Page 20, Line 14. "But it seems possible that CaOx from druses". "from" may be redundant.

ok, deleted.

22. Page 22, Line 19. Here needs a reference for the "randomly distributed" nature of E. senticosus.

Encephalartos senticosus is not directly used for comparison with fossils, thus inclusion of an image seems unnecessary and confusing.

23. Page 24, Line 9. As there is no EDX on the surrounding material, I suggest separating this sentence into two. One is about EDX results, and one is about the contrast in the BSE image.

EDX analysis of surrounding material is included.

24. Page 24. Line 14. A complete sentence is better.

Done

Figures
25. Figure 2I. You can consider adding a label to mark the epidermis.

Done

26. Figure 3A. The specimen number (PB 2021-5-LS) is not given.

ok, included

27. Figures 5B and 6. you can consider marking the "venation" in the image.
28. Figure 7A, B. You can consider marking the "parenchymatic tissue".

Discussion
29. Page 26, Line 25. "Contain lower concentrations of Ca and less common CaOx" needs a reference.

Reference included.

30. Page 27, Line 3. Here needs a reference.

ok; references included

31. Page 27, Lines 13 and 14. After the species names, you can consider adding references to your figures (Fig. 6A-E, Fig. 7).

Done

32. Page 27, Line 18. You can consider adding a reference for the "phytoliths".

Ok; specified as silica grains.

33. Page 27, Line 22. "In another sample". It is not clear which sample is talked about.

Ok; specified that the sample is not shown in results.

34. Page 28, Line 5. You can consider specifying how the former CaOx druses "resemble" those of Encephalartos.

Done

35. Page 28, Line 8. "Tertiary:". The format of this paragraph is different from the others.

Done; format corrected

36. Page 28, Line 15. The "brown organic material" needs a reference.

‚Fig. 9F‘ included

37. Page 29, Line 13. You can consider adding references to figures for the different compositions.

Done

38. Page 29, Line 16. You can show the "identical or different" compositions of the refilled druses and the sediments in the form of a table.

Seems not helpful, due to high variability.

39. Page 30, Line 4. "In a particular sediment matrix". A period seems missing.

Done

References
40. The citation has no number in the text, but the references are listed numerically instead of alphabetically.

**Reviewer #2:** This manuscript aims to document the historical distribution of granular structures made of calcium oxalates in leaves, by studying them in both fresh leaves and fossil leaves (from the Devonian to the Eocene). Although calcium oxalates are well-known in extant plant, their fossil record is more cryptic and this study is potentially shedding light on interesting microstructures resembling that of CaOx druses, in a variety of fossil leaves.

There are, however, limitations that impede immediate publication and I hope that the authors will find my comments useful.

Major comments
1- Diagnostic characters of CaOx druses lately replaced; I do not really understand the characters you use to recognize ghosts CaOx druses from other structures, and a clarification would help the reader. In particular :
(i) from the introduction, it comes out that form, size, density and locations of CaOx druses may vary greatly from one leave to another while you’re stating in the results (page 12 line 11) that ‘The identification of CaOx casts or traces in fossil leaves relies on identifying similarities in the morphology and distribution of these traces’. But then, you’re describing very different structures: ‘bright patches’ of iron oxides (Page20 line 6), spherical organic granules and framboïdal pyrite (Figure 3).

Explanation: Indeed, CaOx occurs in variable forms such as large and small crystals, spherical druses, needles and so on. Fortunately, relatively large druses occur typically in gymnosperms (except most conifers), such as Cycadophyta, and in many dicotyledones. This spherical shape and the distribution patterns, e.g., along the veins, are the basic factors for the recognition. However, it requires the knowledge of CaOx distribution patterns in extant plants. Identification of casts from different CaOx forms such as crystals or raphides would require excellently preserved fossils, and the use of most-suitable examination techniques, without the usual restrictions for museum collections.

(ii) you mention that in addition to CaOx druses, silica may be present, either as ‘mineralization’ in monocotyledons or as crystal sand in gymnosperm; in parallel, in your results ‘ghosts’ of CaOx druses contain silicon. So how do you recognize CaOx druses latter substituted by silica from original silica? Do you expect such original silica to be instable as well when entering the fossil record? Please provide reference if it is the case.

Bio-silica is rare in most of the plant groups relevant for this study (gymnosperms, dicotyledoneous deciduous trees), but many of them contain plenty of CaOx, which is usually not more existent after fossilization. Silicification of fossil plants with silica from the environment is found in numerous cases and can preserve finest structural details, due to its amorphous structure; well known from silicified wood.

(iii) You indicate (page14) that CaOx druses are substituted by pyrite. However, framboidal pyrite can grow on leaves without being a replacement of CaOx druses: see Rickard, D., Grimes, S., Butler, I., Oldroyd, A., & Davies, K. L. (2007). Botanical constraints on pyrite formation. Chemical Geology, 236(3-4), 228-246. So what are your points to claim that they are characteristics of former CaOx druses, and not the result of a simple precipitation onto the surface?

The occurrence within the fossil leaf, in the remnants of the parenchyma, is the main indication for its origin from a spherical bio-structure.

2- Chemical analysis; For extant leaves (Figures 1 and 2), there is no evidence showing that the crystals observed by SEM are CaOx. Elemental analysis is needed.

Element spectrum is added. The identity of CaOx - either mono-hydrate or di-hydrate - crystals has been verified in numerous studies using various analysis methods including Raman spectroscopy, X-ray diffraction, chemical identifications, solubility tests. Other biominerals (CaCO3, Ca-phosphates, CaSO4) usually have different morphology, but rarely well-developed crystal facettes.

For fossil leaves, can you please provide systematically an analysis of the structures and of the hosting rock? For instance, you state for Figure 3F that the dark appearance of the granules and their EDS spectrum indicate an organic deposition, but the matrix just above is also really dark. Does it contain carbon? Finally, most of the EDS spectra lack elemental attribution to peaks. Please ensure that they are all labelled.

Element spectrum peaks have been labelled.

3- Introduction; I think the introduction lacks some crucial information, which impedes a clear understanding of the results and discussion, typically: about how druses form, or about raphides. Similarly, in your results, you present some microstructures as ‘granules’ while they look more like flat 2D deposits… On the contrary, moving Fig1 and 2 from the introduction to the results section will allow you to compare more directly the extant and fossil records. A re organization of your paragraphs may also be beneficial: page 4 is about your previous publication then page 5 is about results from the figure 2 then, the goal of the present study is reported, and finally you go back to your previous study…

Ok; we have re-arranged the Introduction and Discussion according to the Revievwer‘s suggestions

4- A careful edition and a consistency review throughout the MS is necessary. Here are several points, but the manuscript would benefit from a larger check:
- two abstracts in one

Ok; Double abstract deleted

- Fossil either cited as: fossil ,PB 2021-1-LS’ vs. PB2021-5-LS vs. sample ,Gottingen 3480’
- Figures not cited in right order (idem for legend: E after F in figure 7, C between A and B Figure 3, …) or not cited in the text (Figure 5A)
- Write species in italics
- Table 1 incomplete?

Ok; missing data included

I was also wondering if they are supporting files as you wrote at the beginning 'All relevant data are within the MS and its supporting information files'. I couldn’t find them…

We were able to avoid ‚Supporting information files.

Page-by-page comments
- Page 4, line 2: carbonates are among the few main minerals involved in preservation of organisms (with phosphate, silica, …). How can CaCO3 be instable while allowing exceptional preservation? You need a reference here, or explain further.

Ok; specified ‚fossilization of plants‘. As mentioned, most of our leaf fossil samples did not contain any CaCO3 or CaOx; perhaps due to acidic environment during fossilization or decay.

- Page 4 line 13: are you referring to extant or fossil leaves?

Extant leaves. Ok; corrected.

- Page 4 line 19: are you referring to extant or fossil leaves? Why that particular focus on silica?

Species rich in silica phytoliths usually contain rarely or no CaOx. That seems a useful information for the interpretation of structures in leaf fossils.

- Page 6 line 21-26: I don’t understand if those are results from your previous work or from here.

Ok; corrected.

- Page 9: ‘The material derived from several institutes and museums’ => in Table 1 they all appear as coming from the Goldfuß-Museum, University of Bonn. They are no mentions of the collections from Mainz, Göttingen… latter mentioned in the results. Please update the Table. Please also include a column specifying the depositional conditions for each fossil site (are they consistent?) and another for gymno/angiosperms (since you present the results through that property; + monocotyledons vs. dicotyledones if angiosperms).

Ok; missing data are included in Table 1

- Page 10: how many leaves of each specie did you study? How were the leaves stored until analysis? Is there any chance that pollution (I suppose Bonn is polluted as most cities in Western Europe) may affect CaOx druses formation/ extant?

We studied leaves from >200 species in order to get an idea of the distribution patterns of druses and crystals, but not for statistics. When the microscope images were consistent with the overall results, then the images of a single leaf could be sufficient. Many species were studied multiple times with different methods. However, the images show examples of druse distributions, but not complete statistics on concentration and size.

- Page 11: can you add a word about data treatment of CT images?

Done.

- Page 11 line 15: cleaning with? If a chemical, how can you be sure that it did not alter your sample?

‚cleaning‘ replaced by ‚removal of dust or contaminations‘.

- Page 11 line 23: did you perform a control to assess that your preparation is not somehow changing the CaOx druses (chemistry, habits, …)? If not, can you please discuss it?

Possible preparation artefacts are discussed in the cited Reference ‚Ensikat et al., 2023‘.

- Page 14 Line 8-9 : ‘two patterns of granular traces were observed in the leave’: you mean distribution pattern?

Yes; ‚distribution‘ included

- Page 14 Line 9 ‘pyrite-rich granules’: aren’t them made completely of pyrite? Please note that SEM-EDS doesn’t allow you to claim that the granule is made of pyrite (it can be an iron monosulfide).

description modified: ‚rich in Fe and S, likely pyrite‘.

- Page 14 Line 10 Fig 3B cited after 3A
- Page14 Line 15: fig 3 D/ is it Fig 3C?

Yes, indeed. Changed to ‚3 C‘.

- Page 14 line 25: ‘Fig 3D show regularly distributed globular structures’. Can you please point them? I see flat structures irregularly shaped.

Stated more precisely: ‚show some of the regular distributed ..‘

- Page 16 it is strange to have a description of the stratigraphy of Bear Island deposit but not of the other deposits

The large collection of Bear Island fossils is located in our institute in Bonn, thus all the data are available.

- Page 17, Line 1: have you analyzed the particles and are they all made of pyrite? Iron sulfates (jarosite for instance) can also form yellow deposit.

yes, they were analysed; description modified: ‚rich in Fe and S, likely pyrite‘.

- Page 24 line19: ‘white granules and the surrounding white layer consist of silica’ : there are no chemical data of the sediment.
- Page 24 line 22: ‘brown granules of similar size’: and? Made of?

included: ‚which were not yet analysed‘

- Page 26: how your findings feed into an improved understanding of the functions of CaOx?

Our studies are at the beginning. When more data on traces of biominerals in fossils are available, they may be useful to support assumptions about evolution of Ca and Si metabolism and functions of biominerals.

- Page 29 line5 : ‘largely age-independent’ ; you can only say that if all other parameters are fixed, for instance the preservation type of the fossil deposit.

Ok; replaced by ‚the preservation of former CaOx crystal casts is not limited to younger periods.‘

- Page 29 line 14-15 redundant with line 18-19

Line 14-15 deleted

- Page 30 line 3 ‘The type, grain size, and composition of the sediment surrounding the CaOx casts are the main factors in recognizing the casts in fossil leaves in a particular sediment matrix’. So you recognize a cast of CaOx by its surrounding sediment?

‚Type‘ replaced by ‚granular pattern, texture‘. Indeed, in some cases the different composition is useful for the recognition; in cases of homogeneous composition the casts can be visible by characteristic morphology or texture of the grains.

Figures:
- ensure acronyms are explained in the caption.
- When proposing detailed SEM images, please provide an insert showing the location they come from.
- Figures 1, 2, 6: please point arrows to interesting features and anatomical details you are referring to.

Done

- Figure 4: nice pictures!
- Figure 7: which ones are fossil, which one are extant leaves?

Explanation included in Caption

- Figure 8A: what about the granular structures on the bottom left (close to the scale)? Could the hole just above be a cavity left by CaOx?

Yes; that would fit with our explanation

Side notes:
Note that if your samples contain framboidal pyrite, ‘cleaning’ them with water will probably promote oxidation, endangering the conservation of your fossils. (page14)

6. PLOS authors have the option to publish the peer review history of their article ([what does this mean?](https://journals.plos.org/plosone/s/editorial-and-peer-review-process#loc-peer-review-history)). If published, this will include your full peer review and any attached files.


If you choose “no”, your identity will remain anonymous but your review may still be made public.


**Do you want your identity to be public for this peer review?** For information about this choice, including consent withdrawal, please see our [Privacy Policy](https://www.plos.org/privacy-policy).

Reviewer #1: No

Reviewer #2: No
