# Supplementary material for: Detection of traces of calcium oxalate druses in fossil leaves of angiosperms and gymnosperms from different sites and geological periods
Source: PLoS One. 2025 Dec 17;20(12):e0332423. doi: 10.1371/journal.pone.0332423 (PMC12711041; doi:10.1371/journal.pone.0332423)
Supplement: Attachment — Submitted filename: Response to Reviewers.docx [file pone.0332423.s002.docx]

Response to Reviewers

**(Answers and explanations of the authors are in green color)**

PONE-D-25-01413

The evolutionary history and fossil record of calcium oxalate druses in fossil leaves of angiosperms and gymnosperms.

PLOS ONE

Dear Dr. Malekhosseini,

Thank you for submitting your manuscript to PLOS ONE. After careful consideration, we feel that it has merit but does not fully meet PLOS ONE’s publication criteria as it currently stands. Therefore, we invite you to submit a revised version of the manuscript that addresses the points raised during the review process.

Please submit your revised manuscript by May 17 2025 11:59PM. If you will need more time than this to complete your revisions, please reply to this message or contact the journal office at [plosone@plos.org](mailto:plosone@plos.org). When you're ready to submit your revision, log on to https://www.editorialmanager.com/pone/ and select the 'Submissions Needing Revision' folder to locate your manuscript file.

Please include the following items when submitting your revised manuscript:

- A rebuttal letter that responds to each point raised by the academic editor and reviewer(s). You should upload this letter as a separate file labeled 'Response to Reviewers'.
- A marked-up copy of your manuscript that highlights changes made to the original version. You should upload this as a separate file labeled 'Revised Manuscript with Track Changes'.
- An unmarked version of your revised paper without tracked changes. You should upload this as a separate file labeled 'Manuscript'.

If you would like to make changes to your financial disclosure, please include your updated statement in your cover letter. Guidelines for resubmitting your figure files are available below the reviewer comments at the end of this letter.

If applicable, we recommend that you deposit your laboratory protocols in protocols.io to enhance the reproducibility of your results. Protocols.io assigns your protocol its own identifier (DOI) so that it can be cited independently in the future. For instructions see: <https://journals.plos.org/plosone/s/submission-guidelines#loc-laboratory-protocols>. Additionally, PLOS ONE offers an option for publishing peer-reviewed Lab Protocol articles, which describe protocols hosted on protocols.io. Read more information on sharing protocols at <https://plos.org/protocols?utm_medium=editorial-email&utm_source=authorletters&utm_campaign=protocols>.

We look forward to receiving your revised manuscript.

Kind regards,

Borja Cascales-Miñana
Academic Editor
PLOS ONE

Journal requirements:

When submitting your revision, we need you to address these additional requirements.

1. Please ensure that your manuscript meets PLOS ONE's style requirements, including those for file naming. The PLOS ONE style templates can be found at

https://journals.plos.org/plosone/s/file?id=wjVg/PLOSOne_formatting_sample_main_body.pdf and

<https://journals.plos.org/plosone/s/file?id=ba62/PLOSOne_formatting_sample_title_authors_affiliations.pdf>.

2. In your manuscript, please provide additional information regarding the specimens used in your study. Ensure that you have reported human remain specimen numbers and complete repository information, including museum name and geographic location.

If permits were required, please ensure that you have provided details for all permits that were obtained, including the full name of the issuing authority, and add the following statement:

'All necessary permits were obtained for the described study, which complied with all relevant regulations.'

If no permits were required, please include the following statement:

'No permits were required for the described study, which complied with all relevant regulations.'

For more information on PLOS ONE's requirements for paleontology and archeology research, see <https://journals.plos.org/plosone/s/submission-guidelines#loc-paleontology-and-archaeology-research>.

3. In the online submission form, you indicated that [The data that support the findings of this study are based on microscopic images which are archived in the Microscopy/SEM facilities of the Bonn Institute of Organismic Biology (BIOB), University of Bonn, Nussallee 8, 53115, Bonn, Germany. Images are available on request.

Plant collection statements

All plant samples collected in this study were taken from species cultivated in the Botanical Garden, Bonn. This sample collection complies with relevant institutional, national, and international guidelines and legislation.].

All PLOS journals now require all data underlying the findings described in their manuscript to be freely available to other researchers, either 1. In a public repository, 2. Within the manuscript itself, or 3. Uploaded as supplementary information.

This policy applies to all data except where public deposition would breach compliance with the protocol approved by your research ethics board. If your data cannot be made publicly available for ethical or legal reasons (e.g., public availability would compromise patient privacy), please explain your reasons on resubmission and your exemption request will be escalated for approval.

Additional Editor Comments:

Your paper although interesting and scientifically relevant presents a series of points that should be addressed before an eventual acceptance. Please, revise your MS according to received comments to prepare an improved version of your work.


[Note: HTML markup is below. Please do not edit.]

Reviewers' comments:

Reviewer's Responses to Questions

**Comments to the Author**

1. Is the manuscript technically sound, and do the data support the conclusions?

The manuscript must describe a technically sound piece of scientific research with data that supports the conclusions. Experiments must have been conducted rigorously, with appropriate controls, replication, and sample sizes. The conclusions must be drawn appropriately based on the data presented.

Reviewer #1: Yes

Reviewer #2: Partly

2. Has the statistical analysis been performed appropriately and rigorously?

Reviewer #1: N/A

Reviewer #2: N/A

3. Have the authors made all data underlying the findings in their manuscript fully available?

The [PLOS Data policy](http://www.plosone.org/static/policies.action#sharing) requires authors to make all data underlying the findings described in their manuscript fully available without restriction, with rare exception (please refer to the Data Availability Statement in the manuscript PDF file). The data should be provided as part of the manuscript or its supporting information, or deposited to a public repository. For example, in addition to summary statistics, the data points behind means, medians and variance measures should be available. If there are restrictions on publicly sharing data—e.g. participant privacy or use of data from a third party—those must be specified.

Reviewer #1: Yes

Reviewer #2: Yes

4. Is the manuscript presented in an intelligible fashion and written in standard English?

PLOS ONE does not copyedit accepted manuscripts, so the language in submitted articles must be clear, correct, and unambiguous. Any typographical or grammatical errors should be corrected at revision, so please note any specific errors here.

Reviewer #1: Yes

Reviewer #2: Yes

5. Review Comments to the Author

Please use the space provided to explain your answers to the questions above. You may also include additional comments for the author, including concerns about dual publication, research ethics, or publication ethics. (Please upload your review as an attachment if it exceeds 20,000 characters)

Reviewer #1: This study extensively studies the granular structures in plant leaves of different geological ages and makes morphological and distributional comparisons with extant leaves to support the interpretation of CaOx druse remains. The figures are informative and the text has been improved from the last version. However, the logic of the CaOx druse interpretation still could be more quantitive, and a clearer conclusion and more discussions supporting the conclusion are required. So I suggest a major revision.

Major issues:
Abstract:
1. The Abstract is worth a better beginning sentence. The authors can consider to stress the physiological importance of CaOx in plants, and possibly their application for taxonomy, paleophysiology, or paleoenvironmental reconstruction. These would let readers know more about the importance of studying fossil CaOx.

Answer: We have added more details about biominerals and CaOx here and in the Introduction.

2. At the end of the Abstract, the conclusion is still not clear. My comment in the last version was put here directly. I guessed that the authors want to conclude something like (1) “the early and extensive presence of CaOx in fossil plants” and (2) “the diverse chemical composition of former CaOx druses in fossils”. Whatever, the authors are expected to say out directly in the Abstract.

Answer: We have set the focus more on the recognition of biomineral (CaOx) traces.

Introduction:
3. The references of previous studies are scattered over several paragraphs (Page 4 Line 100, Page 5 Line 113, Page 5 Line 120, Page 6 Line 136, Page 6 Line 145), please organize them together.
4. The sequence could be better. I suggest:
(1) previous studies;
(2) the previous studies are used to draw the interpretation bases of this study--“Traces of CaOx druses can be confidently identified due to their… (Page 6, line 139)”, “It has to be accentuated, that morphology, size, density and distribution pattern are verifiable characters of fossil CaOx druses (Page 5 Line 110)”;
(3) next begins with the motivation “Until now, traces of CaOx druses have only been…” (Page 5 Line 116);
(4) then what the authors did “In the present study we examined a variety of fossil leaves from a range of fossil sites from the Devonian to the Eocene… (Page 6, Line 132)”;
(5) at last, the questions to be answered (Page 7 Line 154).

Answer: Thanks for the valuable suggestions. We re-arranged the Introduction accordingly.

Figures:
5. I found that Fig. 1G is a mirror of the insert of figure 1C in Malekhosseini et al. (2022). It is better to replace or remove Fig. 1G.

Answer: We have replaced Fig. 1G.

6. Where the enlarged figures were taken are not labelled from the fossils/leaves, this may raise questions about reliability of the figures.

Results:
7. The paragraph (Page 12 Line 265 to 270) about identification bases seems not part of the result. Consider moving to the Method section.

Answer: Ok, done.

8. In each collection, morphological observations, EDX analyses, and interpretation are mixed. Consider making them into separate paragraphs. The discussion about the preservational processes can be put together in the Discussion section.
9. Generally, the logic of CaOx interpretation is not clear and therefore not convincing enough. Consider (1) making statistics of the granular size (with count numbers, mean values, and standard deviations) of extant and fossil specimens for direct comparison, (2) clarifying the distribution pattern and stressing the shared features with extant leaves.

Answer: We have included far more details about the granular structures, their sizes, composition and distribution, in the Results and additional Table 2.

Discussion:
10. Generally, the discussion about function has a weak connection with the observed results, especially the paragraph about CaOx raphides (Page 22 Line 514 to 523), which have not been reported here. I suggest the authors discuss the early emergence of CaOx druses, which may indicate that early plants have developed CaOx to defend feeding and to regulate calcium as in living plants.

Answer: Yes; we made substantial changes in the Discussion according to these suggestions.

11. Page 23 Line 547, the conclusion that ‘the composition … don’t follow any clear trends’ is not obvious enough to me. Consider adding a summarized table or analyses to support this conclusion. Alternatively, the authors could consider only concluding the “diverse” feature of the preservation.

Answer: Indeed, the composition of the casts appears as variable as the composition of the surrounding sediments. We have written this statement more clearly.


Minor issues:
Abstract:
1. Page 2 Line 37, Page 6 Line 134, it could be better if not to use ‘traces of CaOx druses’ before the interpretation. Using ‘granular structures’ seems better.

Answer: Ok; done.

2. Page 2 Line 43, “the relationship needs to be further summarized”, why not summarize it here?

Answer: ok; „summarized“ replaced by „investigated“.

Introduction:
3. Page 3 Lines 56 to 71, ‘different’ and ‘diverse’ are used many times here. I suggest the authors to summary the recognizable features (size, distribution, …) here instead of stressing the diversity, as they are the vital bases for the following interpretation as CaOx druses.

Answer: Yes, we have added more details in the Introduction.

4. Page 3 Lines 65 to 67, this sentence could be moved forward to be before “the actual mechanisms…”.

Answer: Ok; done.

5. Page 6 Lines 146 to 153, how did the ‘general scenario’ been developed before the results?

Answer: It is a scenario (hypothesis) introduced in a previous publication. We have rewritten this sentence more precisely.

Figures:
6. ‘Angiosperms’ and ‘Dicotyledons’ (Page 4 Lines 82 to 83) need not be capitalized.
7. Page 4 Line 87, ‘to 80 μm’ may be ‘up to 80 μm’.

Answer: Points 6 and 7: done.

8. Page 4 Line 92, (K), how to distinguish CaCO3 from CaOx crystals?

Answer: The identity of the crystals has been verified multiple times with different methods, including Raman spectroscopy, solubility in weak acids, by us and other researchers.

9. Page 7 Lines 164 to 165, ‘the thick cell walls’ seems not related to the topic of this paper.

Answer: We think, it is worth to note that thick cell walls in certain leaves may be the cause for fossil leaves that consist of relatively thick carbon layers.

10. Page 7 Line 169, ‘regular druses leaf lamina’, here needs a preposition.

Answer: Ok; done.

Methods:
11. Page 11 Line 228, the micro-CT method usually reports voltage, current, and voxel size, rather than proprieties of the sensor plate.

Answer: Ok; done.

Results:
12. Page 13 Line 296, this sentence is repeated with Line 281.

Answer: This sentence and images of conifers deleted.

13. Page 17 Line 397, ‘(Figs. 2A, 7E)’, but no “7E” is found.

Answer: „7E“ replaced by „6E“.

14. Page 18 Line 415, ‘along the leaf venation’, it is hard to see in Fig. 6A. If hard to improve the tomographic signal, consider labelling the venation as in Fig. 6D.

Answer: Arrows included.

15. Page 19 Line 449, it is confusing what ‘colourless’ refers to.

Answer: colorless replaced by pale.

16. Page 20 Line 473, ‘visible by LM’, but no LM images in Fig. 9 A, B.

Answer: we preferred the SEM images due to better clarity.

Discussion:
17. Page 23 Line 554, ‘quite early or much later before or after’ is a confusing phrase.

Answer: Ok; sentence re-written.

Other issues:
The grammar and spelling of the text need to be checked.

Reviewer #2: This manuscript presents a description of various putative calcium oxalate druses, in fossils
from the Devonian to the Neogene. Generally, this work is interesting but a clearer statement
of the goal and a summary of the results have to be done, to improve the paper.
Major comments:
1- Title and goal
„The evolutionary history‟ mentioned in the title is not reflected by discussion in the paper. I
would recommend changing the title to „the temporal/ historical distribution‟ rather than the
„evolutionary history‟. This goes along better with the general motivation of the study, which
appears to be the „identification and correct interpretation of CaOx structures‟, according to
an authors‟ response to reviewer 1. This aim must be stated more clearly, in addition to the
questions raised by the authors.

Answer: We have included more information about plant systematic and evolutionary aspects in Introduction and particularly Discussion, so that the title seems to be justifiable.

2- Accordance data - conclusions
The features used to recognize CaOX druses are varying across the paper: shape distribution
texture p 23 vs. granular shape, texture, grain size, composition of sediment surrounding p 24
vs. morphology and distribution p12 vs. distribution and texture later in the same page. This
lack of consistency clearly impairs the paper. Please clearly state them in the introduction or
at the beginning of the results.

Answer: We have included a clear description of the features used for characterization of CaOx traces in the Results chapter. Further changes in Introduction and Discussion should make the text easier to understand.

A table summarizing the results obtained from all your fossils with the selected diagnostic
features will certainly help supporting your hypothesis and increase the clarity of the paper. A
new paragraph in the discussion, highlighting your arguments of interpretation and ruling out
other possibilities (see later paragraph about framboidal pyrite) will also be of critical use for
the audience, to interpret correctly comparable structures in other fossils.

Answer: We have included Table (Table 2) summarizing the results.

3- Previous reviews
It is troubling that part of previous remarks by reviewers was addressed only partially or not
at all, without explanation. Please see further some of them that, in my point of view, were not
answered but must be addressed before publication.
Minor comment:
- Figures 1 and 2 are mentioned and explained in both the introduction and results.
Please only do it once.

Answer: We have deleted several repeated descriptions. However, we are convinced that some explanations in the Introduction and in the begin of the Result section are helpful to better understand the contents.

- Figures are not cited (8C) or not in order (8F before 8E in legend)

Answer: it is corrected.

- The energy range of all your EDS spectra is either blurred or truncated. In Figure 2I
the annotations added are also blurred. Please enhance the quality of such features.

Answer: In most cases, small letters appear blurred due to file compression in pdf; original tif-images are sharp. Others are corrected.

- Results are intertwined with interpretation. Since you chose to have two separate
sections, please remove the latter from the results section (for instance: l.312, l.316,
l.370, l. 487

Answer: In some cases, we think that some explanation in the Results section make it easier to understand.

- Use of contractions and incomplete sentences must be avoided.
- They are redundancies :
compositions of CaOx cast varying; l.42 and 45
„leaves of many species, particularly dicotyledons, CaOx crystals‟ l.55 and 58;
CaOx forms l.56 and 68
„has been substituted […] surrounding sediment‟ l.312-313 and 316-317
- Back and forth within a paragraph of a same notion, with sometimes contradictory
data: „all parts‟ l.54 then „mainly intra-vacuolar membrane‟ l.66; „60-100μm diameter‟
l.280 vs. „30-80μm‟ l.292
- Typos: remove capital letter after „:‟ : l.147, l. 307

Answer: All these mistakes have been corrected.

Page by page review:
l.180: Delete sentence, it is already stated Table 1.

Answer: Details included.

l. 222: „the tescan SEM has low-vacuum capacity‟. More importantly, has it been used?
Otherwise, delete.

Answer: Details included. Low-vacuum mode has been used for fossil samples.

l.308 + legend Figure 3: these are “framboids” not „granules‟. The term is very specific and
you should use it. See Rickard, 2021. Framboids.
<https://doi.org/10.1093/oso/9780190080112.001.0001>

Answer: corrected in text and figure legend. Thanks for these hints.

l.326: if the scale is about 100μm, the structures are either 40 or 70 μm… Please check.

Answer: Scale is correct. Text corrected.

l.355: Pseudobornia ursina not mentioned Table 1

Answer: It is not included in Table 1 because no data or images are used.

l.375: no analyses given to evidence the Si-Al nature of the sediment
l.474: iron is not mentioned while visible on EDS spectrum. Same l. 495.

Answer: corrected. Additional spectra included.

l.549: CaSO4 has never been mentioned before. Can you explain?

Answer: Details on CaSO4 in granular structures are included.

Comments on previous reviews:
Reviewer 1 + Reviewer 2
R1 : 15. Page 16, Line 19-21. “This formation…Røedvika Formation (Horn & Orvin, 1928)”.
The stratigraphy study's history is irrelevant to the specimen's age and the paper's topic.
Not addressed.
R2 : - Page 16 it is strange to have a description of the stratigraphy of Bear Island deposit but
not of the other deposits
The large collection of Bear Island fossils is located in our institute in Bonn, thus all the data
are available.
But it is still irrelevant to the paper. Please delete.

Answer: Ok; deleted.

R1 : 15. Page 29, Lines 6-7. "Differences in fossilization conditions in various sites". This
aspect of information was not shown in the paper. You can consider adding this information
to a summarizing table.
‚the most influencial and limiting„ deleted; so the statement seems to be correct
R2 :- Page 9: „The material derived from several institutes and museums‟ => in Table 1 they
all appear as coming from the Goldfuß-Museum, University of Bonn. They are no mentions
of the collections from Mainz, Göttingen… latter mentioned in the results. Please update the
Table. Please also include a column specifying the depositional conditions for each fossil site
(are they consistent?) and another for gymno/angiosperms (since you present the results
through that property; + monocotyledons vs. dicotyledones if angiosperms).
Ok; missing data are included in Table 1
The last sentence has not been addressed. Since you mentioned fossilization conditions in
your discussion, the role of depositional conditions could be further extended, and references
provided.

Answer: A detailed inclusion and discussion of the fossilization conditions for this heterogeneous group of samples would be very speculative. This may be useful for detailed studies of fossils from well-known Lagerstätten. Here we refrain.

Reviewer 1
14. The "Fossil record of CaOx cast preservation" section does not provide much more
information than the Results section. You can consider making a table to summarize these
granular structures' distribution, morphology, and chemistry from different species, ages, and
localities.
Ok; we have deleted most of the redundant information and re-arranged the Discussion.
The table asked would be very useful.
38. Page 29, Line 16. You can show the "identical or different" compositions of the refilled
druses and the sediments in the form of a table.
Seems not helpful, due to high variability.
Which is the very reason why a table will help gain in clarity !

Answer: We have included a Table (Table 2) summarizing the results.

13. Page 14, Line 7. "Iron oxide caused by storage in glycerol" needs a reference.
Additional information or images on these contamination seem unnecessary and confusing.
I do not agree with that. Glycerol is supposed to store specimens away from the atmosphere,
thus avoiding oxidation by dioxygen or humidity. To me, it is quite surprising that storage in
glycerol causes oxidation. So please delete or add a reference as requested.

Answer: We think, this fact is too important to delete it. We have added some details and an image of the contamination.

Reviewer 2
Major comments
1- Diagnostic characters of CaOx druses lately replaced; […]
(i) from the introduction, it comes out that form, size, density and locations of CaOx druses
may vary greatly from one leave to another […]
Explanation: Indeed, CaOx occurs in variable forms such as large and small crystals,
spherical druses, needles and so on. Fortunately, relatively large druses occur typically in
gymnosperms (except most conifers), such as Cycadophyta, and in many dicotyledones. This
spherical shape and the distribution patterns, e.g., along the veins, are the basic factors for the
recognition. However, it requires the knowledge of CaOx distribution patterns in extant
plants. Identification of casts from different CaOx forms such as crystals or raphides would
require excellently preserved fossils, and the use of most-suitable examination techniques,
without the usual restrictions for museum collections.
Maybe a clear sentence stating that (i) you compare with the actual record and (ii) you
describe specific distribution/ shape/ size/ textures for each type of plants will help. (see
previous major comment 2) of this review.

Answer: We have described the diagnostic characters more clearly in the introduction.

(iii) You indicate (page14) that CaOx druses are substituted by pyrite. However, framboidal
pyrite can grow on leaves without being a replacement of CaOx druses: see Rickard, D.,
Grimes, S., Butler, I., Oldroyd, A., & Davies, K. L. (2007). Botanical constraints on pyrite
formation. Chemical Geology, 236(3-4), 228-246. So what are your points to claim that they
are characteristics of former CaOx druses, and not the result of a simple precipitation onto the
surface?
The occurrence within the fossil leaf, in the remnants of the parenchyma, is the main
indication for its origin from a spherical bio-structure.
The authors of the paper mentioned above did obtain pyrite crystals within the parenchyma,
which proves that such crystals can be the direct result of precipitation and not due to the
substitution of former CaOx druses as you state. Please bring other points to strengthen your
hypothesis here, or remove these data.

Answer: It seems that Rickard et al. did not mention the locations of CaOx crystals as possible spaces for growth of framboids, after decay of the CaOx. Any available voids may allow their growth, but the size and the spherical shape fit well with CaOx druses.

2- Chemical analysis;
For fossil leaves, can you please provide systematically an analysis of the structures and of
the hosting rock? For instance, you state for Figure 3F that the dark appearance of the
granules and their EDS spectrum indicate an organic deposition, but the matrix just above is
also really dark. Does it contain carbon? Finally, most of the EDS spectra lack elemental
attribution to peaks. Please ensure that they are all labelled.
Element spectrum peaks have been labelled.
The remark has only been partially addressed. Could you please provide EDX spectra of the
sediment for Fig 3D, 3F and 4? Since you have stated the composition of sediment
surrounding as a factor of identification of CaOX druses, this data must be consistently
provided.

Answer: Additional spectra are now included.

Page-by-page comments
- Page 4, line 2: carbonates are among the few main minerals involved in preservation of
organisms (with phosphate, silica, …). How can CaCO3 be instable while allowing
exceptional preservation? You need a reference here, or explain further.
Ok; specified ‚fossilization of plants„. As mentioned, most of our leaf fossil samples did not
contain any CaCO3 or CaOx; perhaps due to acidic environment during fossilization or decay.
This information is interesting and must be discussed and supported by data or references of
the depositional environment of your fossil deposits (in Table 1 for instance), as asked
previously).

Answer: We have indeed examined several fossil leaves in CaCO3 sediment, but the quality of the preservation of parenchymatic structures was very bad and the sediment structure was too coarse, so that the relevant micro-structures were not visible. We have mentioned in the Discussion that many samples had insufficient quality.

- Page 10: how many leaves of each specie did you study? How were the leaves stored until
analysis? Is there any chance that pollution (I suppose Bonn is polluted as most cities in
Western Europe) may affect CaOx druses formation/ extant?
We studied leaves from >200 species in order to get an idea of the distribution patterns of
druses and crystals, but not for statistics. When the microscope images were consistent with
the overall results, then the images of a single leaf could be sufficient. Many species were
studied multiple times with different methods. However, the images show examples of druse
distributions, but not complete statistics on concentration and size.
This is very important as it enhances the representativeness of your data. It should be
mentioned in the material and methods part.

Answer: Statement included as suggested. Contamination of fossil samples has been mentioned in the discussion.

6. PLOS authors have the option to publish the peer review history of their article ([what does this mean?](https://journals.plos.org/plosone/s/editorial-and-peer-review-process#loc-peer-review-history)). If published, this will include your full peer review and any attached files.


If you choose “no”, your identity will remain anonymous but your review may still be made public.


**Do you want your identity to be public for this peer review?** For information about this choice, including consent withdrawal, please see our [Privacy Policy](https://www.plos.org/privacy-policy).

Reviewer #1: No

Reviewer #2: No

[NOTE: If reviewer comments were submitted as an attachment file, they will be attached to this email and accessible via the submission site. Please log into your account, locate the manuscript record, and check for the action link "View Attachments". If this link does not appear, there are no attachment files.]

While revising your submission, please upload your figure files to the Preflight Analysis and Conversion Engine (PACE) digital diagnostic tool, <https://pacev2.apexcovantage.com/>. PACE helps ensure that figures meet PLOS requirements. To use PACE, you must first register as a user. Registration is free. Then, login and navigate to the UPLOAD tab, where you will find detailed instructions on how to use the tool. If you encounter any issues or have any questions when using PACE, please email PLOS at [figures@plos.org](mailto:figures@plos.org). Please note that Supporting Information files do not need this step.

Answer: PACE procedure for Figures carried out.

*In compliance with data protection regulations, you may request that we remove your personal registration details at any time.* [*(Remove my information/details)*](https://www.editorialmanager.com/pone/login.asp?a=r)*. Please contact the publication office if you have any questions.*
