# Supplementary material for: Detection of traces of calcium oxalate druses in fossil leaves of angiosperms and gymnosperms from different sites and geological periods
Source: PLoS One. 2025 Dec 17;20(12):e0332423. doi: 10.1371/journal.pone.0332423 (PMC12711041; doi:10.1371/journal.pone.0332423)
Supplement: Attachment — Submitted filename: Responce to reviewers.docx [file pone.0332423.s003.docx]

**Response to Reviewers**

Reviewers' comments:

6. Review Comments to the Author

Please use the space provided to explain your answers to the questions above. You may also include additional comments for the author, including concerns about dual publication, research ethics, or publication ethics. (Please upload your review as an attachment if it exceeds 20,000 characters)

Reviewer #1: The authors significantly improved the abstract and discussion sections and make a good table to summarise the abundant results observed, according to our suggestions. I found the paper generally meets the publication criteria but there are still some disorganisations in the text. So I suggest acceptance after a minor revision.

Here are some major points:
1. I agree with the Reviewer 2 that the title needs some changes. The ‘evolutionary history’ is not stressed in the paper at all. I have to stress that if you have a title like “the evolutionary history of calcium oxalate druses”, we will expect the reconstruction of the morphological or distributional changes of the druses along the phylogenetic tree.

Answer: We changed the title according the view of both reviewers.

Old title: The evolutionary history and fossil record of calcium oxalate druses in fossil leaves of angiosperms and gymnosperms.

New title:

Detection of traces of calcium oxalate druses in fossil leaves of angiosperms and gymnosperms from different sites and geological periods.

2. As for the newly added Table 2, the authors only listed the sizes. (1) I do not understand why the authors do not make a statistic of the granule sizes, which would significantly help the comparison of sizes; (2) I suggest adding the morphology and distribution patterns to the table as well, which would help to support the interpretation as CaOx druse remains.

Answer: Concerning point (1), we have initiated a new study to measure the size of crystals and druses in different parts of numerous recently collected plants. The results will serve as a reference catalog for comparison with fossil material. Identification of CaOx traces and the inclusion of statistical analyses will require a larger sample of fossil leaves, which is part of our planned future research.

Statistics on the present results appear to be of little use due to the frequent inhomogeneity of the samples.

(2) Answer: We have included the distribution patterns in Table 2.

3. Related to the question (3), a discussion about the variation of composition inside each clade is needed.

Answer: Data on the compositions are shown in the Results, in Table 2, and are mentioned in the Discussion. Further discussions about the variations would be speculations.

4. In the discussion part, I want to see some words to exclude other possible interpretations of the granular structures, e.g. cell nuclei or special mesophyll cells.

Answer: We have mentioned some examples of structures which could form visible structures in fossil leaves that may resemble CaOx casts, but show different distribution or shape, and we mentioned pollen in plant fossils, which also look similar, and the discussion about their differences to CaOx casts in a previous publication.

Some minor points are here:
ABSTRACT:
1. Line 46, “These granular structures resembled patterns”, specify what pattern please.

Answer: „in morphology and distribution“ added.

2. Line 47, “can be interpreted”, you can directly say “were interpreted”.

Answer: Ok, replaced.

3. Line 52, “the correlations still need to be investigated” can be deleted.

Answer: It has been deleted.

INTRODUCTION:
4. Line 62, here the proportion of angiosperms is listed, what about gymnosperms?

Almost all of the studies focused on higher plants and only few on gymnosperms. We have not found comprehensive statistics on the frequency of CaOx in gymnosperms, but we have included our experiences, that almost all Cycads and Conifers have CaOx crystals.

5. Line 109, “the obvious instability” necessarily needs references or detailed explanation!

Answer: Explanation added.

6. Lines 118 – 121, I cannot understand why these patterns are “confident” and “verifiable” identifiers, give references please.

Answer: „confident“ replaced by „reliably“; Reference added.

7. Lines 158 – 160, the sentence “In leaves of …” should not be put here but within the introduction part of living gymnosperm CaOx.

Answer: It has been deleted, as it was redundant.

DISCUSSION:
8. Line 571, “frequently” should be replaced by a certain proportion from your results.

Answer: Examples (Figs. 3-5) are mentioned; „frequently“ replaced.

9. Line 579, need references or images for support.

Answer:
10. Lines 580–581, this description of a previous study should be moved to the introduction part.

Answer: We have deleted this sentence.

11. There are still some spelling mistakes in the text, please check them.

Reviewer #2: Compared to the previous version of the manuscript, the addition of a table and several changes in the figures have improved the quality of the data, enhancing the relation between data and conclusions. The abstract and introduction are also more informative but to my points of views, there are still some points that need to be addressed.

Major issues:

General:
1. For me, the revised manuscript and the revised manuscript with track changes are different! Particularly, the results paragraphs on Eckfeld and Willerhausen collections differ. In my opinion, the ‘revised’ version seems more completed.

Answer: I have checked the files on my computer and could not find such differences. Perhaps a previous working version of the „manuscript with track changes“ had been submitted accidently. We would like to apologize for this.

2. The goal of the authors is much clearer and the work recentered on the recognition of CaOx druses. However, a hook to understand their importance is still missing (for instance in the abstract) and impedes to figure out the significance of your work. It’s unfortunate.

Answer: We have added a sentence about the importance at the end of the abstract.

3. The authors state that pyrite framboids are substitutes of former CaOx druses. This statement is interesting but remains hypothetical: the size of pyrite framboids can go up to several hundreds of micrometers, contrary to the size of granules observed here in the fossil leaves (60 µm max. according to table 2).

Several of the sentences would benefit from a more precautious approach (L321-324 of ‘revised version’ – not in track changes one + L524-525).

Answer: We added some notes in the discussion.

Material:
A response to a former question about the number of samples and specimens observed was directly added at the end of the material paragraph. It seems a bit clumsy and should be rephrased.
Answer: We have mentioned „the inhomogeneity of the samples“ as a difficulty to obtain statistically relevant data.

Results:
Consider moving the paragraph from l.280 to 291 to the method section (as it has been done for a former sentence), since it deals about the methodology of identification. Like this, and although it allows to understand your thought process, it is a bit hard to read, between two pieces of results.

Answer: Ok, we deleted this section, as it was redundant with parts in Introduction and Discussion.

The authors stress that in fossil leaves, ‘there was a high degree of uncertainty in the morphological interpretation’ (l281) while they latter state that ‘spherical morphology of suitable size’ (l294) is among the characteristic trends to identify Caox druses. Perhaps a consensus approach would be to state that in well-preserved fossils, morphology + distribution are the key characteristics but with poor preserved fossils, only the distribution was used?

Answer: Thanks for this suggestion. We have included it.

Discussion:
I recommend moving the second sentence later in the text (after ref. 19 and 20) so the occurrence of druses in old fossil plants stresses their importance in providing information on evolution, etc …
Answer: It has been done

Minor issues
- Typos: ‘whithout’, ‘staightforward’
- L42, compared with biomineral patterns in leaves -> compared with biomineral patterns in extant leaves?

Answer: It has been done

- L48, depends on varying fossilization conditions -> depends on fossilization conditions?

Answer: It has been done.

- L178, leaf fossils -> fossil leaves?

Answer: It has been done

- L458, ‘colour’ has been replaced by ‘composition’. But in the following, you’re definitively talking about colour since you mention ‘pale’ or ‘darker’ patches. Why not ‘by colour and composition (and texture)’?

Answer: Good suggestion; we agree.

7. PLOS authors have the option to publish the peer review history of their article ([what does this mean?](https://journals.plos.org/plosone/s/editorial-and-peer-review-process#loc-peer-review-history)). If published, this will include your full peer review and any attached files.

If you choose “no”, your identity will remain anonymous but your review may still be made public.

**Do you want your identity to be public for this peer review?** For information about this choice, including consent withdrawal, please see our [Privacy Policy](https://www.plos.org/privacy-policy).

Reviewer #1: **Yes:** Dr. Qingyi Tian

Reviewer #2: No
